# Leptomeningeal involvement in B-cell chronic lymphocytic leukemia: a case report and review of the literature

**DOI:** 10.1186/1756-0500-7-645

**Published:** 2014-09-13

**Authors:** Simone Lima de Souza, Fábio Santiago, Marilza de Moura Ribeiro-Carvalho, Adriano Arnóbio, Andréa Ribeiro Soares, Maria Helena Ornellas

**Affiliations:** Departamento de Patologia, Faculdade de Ciências Medicas, Programa de Pós Graduação em Ciências Médicas, Universidade do Estado do Rio de Janeiro, 444/4° andar, Vila Isabel, Rio de Janeiro Brazil; Serviço de Hematologia, Hospital Universitário Pedro Ernesto, Universidade do Estado do Rio de Janeiro, Rio de Janeiro, Brazil

**Keywords:** Chronic lymphocytic leukemia, Flow cytometry, Central nervous system involvement

## Abstract

**Background:**

Central nervous system involvement is considered a rare complication of chronic lymphocytic leukemia, and so there is the risk of being overlooked.

**Case presentation:**

We report a case of central nervous system involvement in a 75-year-old mulatto woman with chronic lymphocytic leukemia after 5 years of follow-up and a literature review on the subject. The clinical course, treatment and outcome are described. A systematic, meticulous and comprehensive analysis of existing publications regarding chronic lymphocytic leukemia with central nervous system involvement was performed.

**Conclusion:**

We concluded that central nervous system involvement of chronic lymphocytic leukemia is probably not associated with any evident risk factors. Diagnostic approach differs by institutions but often includes imaging, morphology and flow cytometry. Resolution of central nervous system symptoms can usually be accomplished with intrathecal chemotherapy or irradiation followed by systemic treatment. The recognition of this entity by clinicians could lead to early detection and treatment, resulting in better outcomes in this rare complication.

## Background

B-cell chronic lymphocytic leukemia (B-CLL) occurs in elderly patients and is characterized by proliferation and accumulation of monoclonal B lymphocytes, expressing CD5, CD20 and CD23 molecules [1, 2]. The involvement of central nervous system (CNS) is considered a rare and aggressive complication. Nevertheless, autopsy studies suggest that it is much more common than supposed in clinical practice [3–5]. Reske-Nielsen and co-workers (1974) were one of the first groups to detect this CNS infiltration. These authors observed neurologic infiltration in 10 advanced stage patients, in a total of 14 CLL necropsies. However, none of them had any neurologic symptoms [6].

In most studies, case presentations are presented [7–10] whereas others emphasize the role of diagnostic methods [11, 12]. Our aim is to present a new case of CLL with leptomeningeal involvement and also to make a review of the literature, focusing studies where immunophenotypic profile was performed.

## Case presentation

A 75-year-old mulatto woman was admitted to the University Hospital with a 2-week history of headache, otalgia in the right ear, fever, dizziness and dysphagia. Physical examination showed diffuse lymphadenopathy, gait instability, peripheral right-side facial nerve palsy and incoordination of lower limbs. Muscle strength and reflexes were normal in the upper limbs. Her medical history was remarkable for hypertension, type 2 diabetes mellitus and chronic lymphocytic leukemia (CLL), diagnosed five years earlier (Rai stage I with evolution to Rai II with lymphocyte increasing and response to treatment). She was no longer receiving treatment for this disorder and the last chemotherapy had occurred five months before. She had been treated before in three occasions, with clorambucil and prednisone, due to lymphocytosis and liver and spleen enlargement, from June to Sep 2005 (4 cycles) from Oct 2006 to June 2007 (8 cycles) and from June 2008 to August 2009 (14 cycles).

Peripheral blood evaluation revealed leucocytosis (white blood cell – WBC = 24.3×10^9^/L), a mild anemia (hemoglobin = 10.5 g/L) and 123×10^9^ platelets/L, and immunophenotypic study confirmed B-CLL. On the biochemical examination, normal values of serum bilirubin, amylase, BUN, uric acid, albumin, transaminases, calcium, potassium and iron were observed. As neurological examination had suggested a possible left brain ischemia, computed tomography (CT) scan of the brain was performed, but it was within normal limits. Magnetic resonance imaging (MRI) was recommended, but the patient refused to do it because of claustrophobia. Upper endoscopy was normal. Acute otitis media was treated by myringotomy (showed serosanguineous fluid) with clavulin and acyclovir.

**Figure 1:**
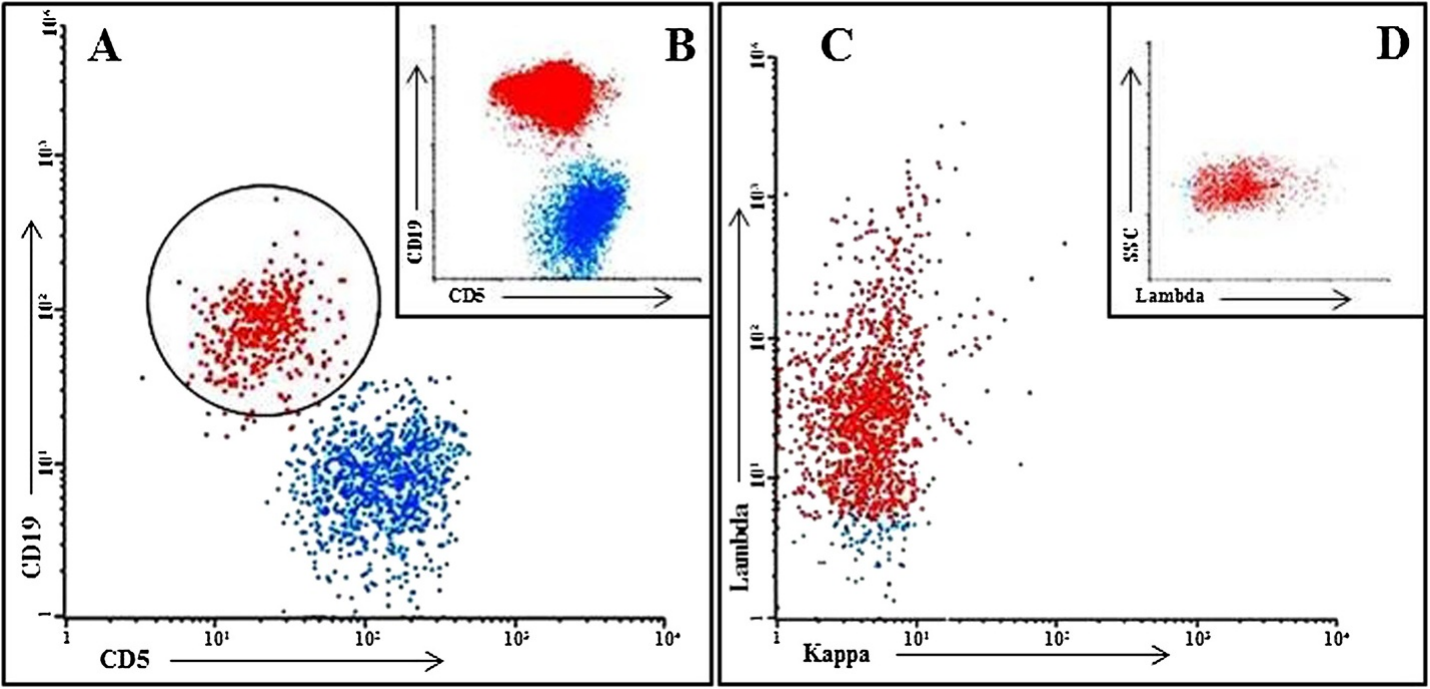
**Cerebrospinal fluid and peripheral blood immunophenotyping: flow cytometric dot plots of cerebrospinal fluid (A, C and D) and peripheral blood (B) demonstrating CLL populations.** The units on the x- and y-axes are fluorescent intensitity. **(A and** **B)** CD5 XCD19 in cerebrospinal fluid and peripheral blood cells, respectively. Four colour cytometry was performed using FACSCalibur (BD Biosciences) flow cytometry. The minority of cerebrospinal fluid cells were positive to double staining showing in circle of the panel **A**. Panel **C** shows monoclonality to Ig lambda.

An immediate lumbar puncture was performed and the cerebrospinal fluid (CSF) revealed WBC count of 18 leukocytes/mm^3^ (mononuclear cells: 80%; polimorphonuclear neutrophils: 20%), glucose of 92 mg/dL and total protein of 50 mg/dL. GRAM, BAAR and criptococcal were negative; bacteria, mycobacteria and fungi cultures; and herpes virus in polymerase chain reaction (PCR)/cryptococcal antigens were negative, VDRL and TPHA negatives. Cytological examination of liquor showed typical and immunophenotypic analysis revealed small and monomorphic lymphocytes subset with profile: CD19^+^, CD5^low^ and lambda^+^, confirming CLL in CSF (Figure 1).

There was complete resolution of neurological symptoms liquor infiltration after 6 doses of weekly intrathecal methotrexate and systemic fludarabine plus cyclophosphamide treatment. She was outpatient followed but died nine months after, due to sepsis. Autopsy was not done.

### Review of the literature of CLL cases involving CNS

The review of the literature was conducted according Cochrane guidelines and Preferred Reporting Items for Systematic Review and Meta-analysis recommendations [13, 14].

In December 13, 2013 the search was conducted in PubMed Central (National Center for Biotechnology Information, US National Library of Medicine, Washington). In order to use search terminology Medical Subject Headings (MeSH) headings was used. We conducted two search strategies.

The MeSH terms used in the database search included: Leukemia, Lymphoid/complications; Leukemia, Lymphoid/pathology; Brain Neoplasms/radiography; Leukemia, Lymphoid/physiopathology; Leukemia, Lymphocytic, Chronic, B-Cell/cerebrospinal fluid; Leukemia, Lymphocytic, Chronic, B-Cell/drug therapy; Leukemia, Lymphocytic, Chronic, B-Cell/radiotherapy; Leukemia, Lymphocytic, Chronic, B-Cell/drug therapy; Lymphoma, B-Cell/radiotherapy; Leukemia, Lymphocytic, Chronic, B-Cell/diagnosis; Central Nervous System Neoplasms/immunology.

For the second search strategy the term "Chronic lymphocytic leukemia" was used in the search titles.

Studies were selected if they met the following inclusion criteria:

1. Indexed articles between January 1^st^ 1975 and December 13^th^ 2013;
2. The search included the follow documents: letters to the editor, case presentations, case series, original research reports and reviews;
3. Clinical research articles in adults;
4. The following idioms were included: English, French, German and Spanish;

For exclusion criteria:

1. Articles published outside of proposed period;
2. Other published articles not specified by inclusion criteria;
3. Experimental clinical research articles;
4. Articles written by languages not specified by inclusion criteria;
5. Ritcher syndrome.

Search results were merged using EndNote X7 (Thomson Reuters, TX, USA) reference manager, and duplicate records removed. The titles and abstracts of the articles were then examined and reports that were not randomized studies and those that were not relevant were removed.

All abstracts were independently reviewed by two authors, and full texts were reviewed for determining eligibility if abstracts were incomplete. Manuscripts that met inclusion criteria were retained for full analysis. Any disagreements were resolved by further discussion involving an additional author.

**Figure 2:**
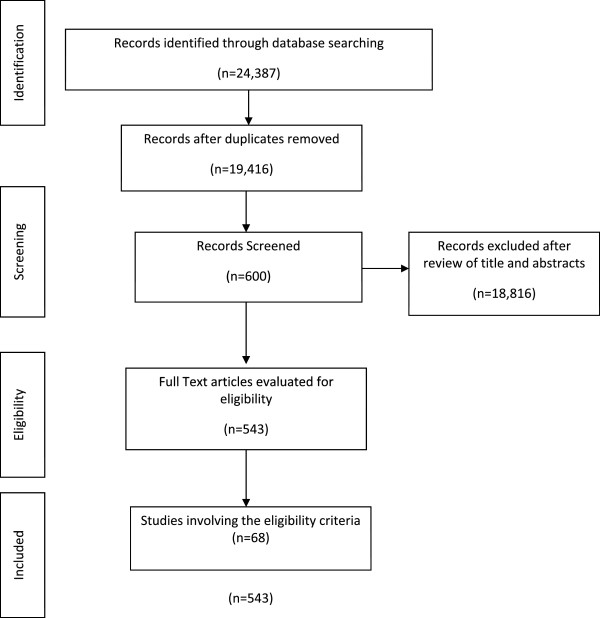
**Flow diagram representing the selection studies.**

The search identified 24,367 articles, of which 4,951 were removed for being duplicated. A total of 68 articles were eligible satisfying established criteria for two research strategies [2, 5, 7–10, 15–79]. The flow diagram representing the selection studies is shown in Figure 2.

The characteristics of the 89 previous reported patients and those of the present case are summarized in Table 1. More men than women were diagnosed with CNS-CLL (male/female ratio: 1.77). The median time for CNS disease was 29.5 months (ranging from 0 to 156 months), since CLL diagnosis. Positive cytology alone, and with immunophenotyping analyses, was positive in 57 of 64 cases (89%). The majority of patients with available clinical data were treated with intrathecal chemotherapy and/or radiotherapy with systemic chemotherapy and most of them demonstrated improvement in their symptomatology. Neurological symptoms at presentation varied. Visual changes occurred in one quarter of cases, with or without other manifestations, such as slurred speech, headaches, gait disorder and memory loss.

**Table 1:** **Patient characteristics (N = 90)**

Data		
**Age, median (range)**	65	(33–89)
Missing data	4	
**Gender, N (%)**		
Male	55	(61)
Female	31	(35)
Missing data	4	(4)
**Stage, N (%)**		
Rai 0,1 and Binet A	34	(38)
Rai 2 and Binet B	16	(18)
Rai 3,4	27	(30)
Missing data	13	(14)
**Time to CNS disease, N (%)**		
Until 1 year	35	(39)
>1 ≤ 5 year	29	(33)
> 5 year	13	(14)
Missing data	13	(14)
**CNS site of involvement, N (%)**		
Leptomeningeal	53	(59)
Leptomeningeal and brain parenchima	4	(5)
Brain parenchima	12	(13)
Others^a^	21	(23)
**Neuroimaging, N (%)**		
Positive	23	(26)
Negative	23	(26)
Missing data	44	(48)
**Cerebrospinal fluid, N (%)**		
Positive	57	(63)
Negative	7	(8)
Missing data	26	(29)
**Histology, N (%)**		
Yes (positive)	15	(17)
No	75	(83)
**Treatment**		
IT + systemic chemotherapy	17	(19)
IT + radiation therapy	13	(14)
IT chemotherapy	13	(14)
IT + systemic + radiation therapy	8	(9)
Systemic chemotherapy	8	(9)
Systemic + radiation therapy	6	(7)
Radiation therapy	5	(6)
None	9	(10)
Not available	11	(12)
**Survival status, N (%)**		
Alive	38	(42)
Dead	38	(42)
Missing data	14	(16)

IT = intrathecal treatment.

^a^hypothalamus, optic nerve, pituitary, skull base thickening, spinal cord, dura mater, epidural.

## Conclusions

Chronic lymphocytic leukemia (CLL) involving the central nervous system (CNS) presents with a high clinical heterogeneity [80]. Some patients experiment a real indolent course and have life expectancy similar to unaffected individuals, while others have an aggressive malignancy that requires early treatment and have limited survival. As the disease progresses, lymphocytes infiltrate lymph nodes, spleen and liver [81–83]. Occasionally, these cells can infiltrate other organs, such as skin [84], lungs, pleura, [85], gonads, prostate [86], kidneys [87] and gastrointestinal tract [88]. Ratterman and colleagues (2014) described in a recent systematic review about extramedullary CLL, that CNS and skin were the most frequently affected sites [81].

Including the present case, 92 patients have been described with CNS-CLL in this review, and it provides the largest summary of patients previously reported. Corroborating other authors’ results, the current analysis suggests that CNS-CLL is not as unusual as it seems [2, 80, 81]. Nevertheless, CNS-CLL may be neglected for several reasons, leading to improper and under-reported diagnosis. Moreover, other neurological manifestations, including opportunistic infections, secondary brain malignancies, therapy-related neurotoxicity, metabolic encephalopathies and paraneoplastic neurologic syndromes, were described in this entity [80]. Given the paucity of reports describing central nervous system (CNS) involvement, large scale investigations are complex and challenging to perform and, consequently, the appropriate identification and management of this disease are hampered.

For the diagnosis evaluation, three parameters are routinely used: (a) clinical signs and symptoms, (b) radiological imaging, (c) cerebrospinal fluid (CSF) analysis. Clinical manifestations ranged from the absence of neurological signs, as described in autopsy series [5, 89], to a diverse and non-specific spectrum of symptoms (Table 1), which are common in this age group. Dementia, gait disturbance, hearing loss and other neurologic symptoms are present in many diseases found in senescence. Studies attempting to identify risk factors in patients who develop CNS infiltration have failed in detecting common predisposing conditions. A relevant number of patients had the CNS involvement confirmation at the same time of chronic lymphocytic leukemia diagnosis. In addition to these *in vivo* findings, autopsy studies confirm that many patients who had CNS involvement never referred neurological symptoms [3–6].

Radiologic findings (computed tomography [CT] and magnetic resonance imaging [MRI]) of CNS involvement have been described as diffuse coating of the leptomeninges by a thin layer of leukemic cells, nodule growths, plaque-like deposits and intraparenchymal infiltration [2, 79]. However, cranial imaging has low sensibility in detecting intracranial CLL, in comparison with other diseases [90] and low specificity, as it may be misdiagnosed with meningioma [15]. The present review confirms previous studies, in which imaging investigation detected CNS infiltration in approximately 40% of CLL cases [2, 80]. Besides, in 4 cases of CSF negative for CLL infiltration, radiologic imaging revealed brain masses [16–19].

Regarding the CSF analysis, the sample is obtained by lumbar puncture and all precautions must be taken in order to minimize contamination by peripheral blood. This evaluation includes global and specific cell count, glucose, total protein, culture and cytology. Although CSF cytology examination is considered the “gold standard” for diagnoses, due to its excellent specificity (>95%), its sensibility is within 50% to 60% [91].

Several variables associated with false negative cytology have been identified [92], mainly due to the paucity of tumor cells in CSF. Also, CLL lymphocytes can not be distinguished from reactive lymphocytes by morphology alone [93]. Reactive lymphocytes may be common in these patients, who present immune suppression and are susceptible to opportunistic infections.

Flow cytometry immunophenotyping (FCI) is an objective and rapid method for qualitative and quantitative analysis of cell suspensions. It can detect small populations of tumor cells with aberrant surface-marker expression, through multicolor and multiparameter analysis. FCI is considered to be two to three times more sensitive than cytology in detecting CSF malignant infiltration [2, 11, 12, 80, 92, 94–96]. It is particularly helpful for the detection of clonal populations of small sized B lymphocytes [97] which would lead to the indication intrathecal chemotherapy and or radiotherapy. The sensitivity of this technique can be enhanced by cytocentrifuge methods as described by Akintola-Ogunremi and coworkers (2002) [7].

Polymerase chain reaction (PCR) also known to improve sensitivity in detecting CSF malignancy. However, PCR assay requires more time than FCI, and needs the selection of specific tumor cell primers. Therefore its routine employment continues to be restricted [98]. Vogt-Schaden and colleagues (1999) confirmed leptomeningeal dissemination in a patient with CLL using a clone-specific CDR3 region in IgH encoding locus. It has already been reported in a study about lymphomas and reactive lymphoid proliferations that the use of both FCI and PCR techniques in different sample specimens [77]. The authors observed a superior sensitivity of FCM when compared with PCR (77% *vs* 64%). However, they concluded that combined use should be considered as the sensitivity reached to 93% [99].

The identification of specific tumor markers for CNS leukemic invasion has remained elusive. Soluble CD27 (sCD27) was already considered a probable marker for leptomeningeal involvement. Van den Bent and coworkers (2002) [100] demonstrated that it was useful in ruling out the disease with a negative predictive value of 92%. Nevertheless sCD27 was also detected in various non-malignant inflammatory conditions (positive predictive value = 54%).

Although larger prospective studies with longer follow-up are required, we suggest that patients should be carefully evaluated for more precise evidence of CNS lymphoid malignancies before receiving toxic treatments. In addition mentioning Pirandello’s masterpiece “So It Is (If You Think So)” clinicians should use all possible diagnostic tools to identify CNS infiltration in CLL. After this systematic review, we can conclude that the early identification of the central nervous system involvement and prompt treatment should be considered to reduce morbidity and improve quality of life of these patients.

## Consent

Written informed consent was obtained from the patient for publication of this Case presentation and any accompanying images. A copy of the written consent is available for review by the Editor-in-Chief of this journal.

## Abbreviations

B-CLL: B-cell chronic lymphocytic leukemia
CD: Cluster of differentiation
CNS: Central nervous system
WBC: White blood cell
BUN: Blood urea nitrogen
CT: Computed tomography
MRI: Magnetic resonance imaging
CSF: Cerebrospinal fluid
FCI: Flow cytometry immunophenotyping
PCR: Polymerase chain reaction
sCD27: Soluble CD27.
